# Responsive Adsorption of *N*-Isopropylacrylamide Based Copolymers on Polymer Brushes

**DOI:** 10.3390/polym12010153

**Published:** 2020-01-07

**Authors:** Guillaume Sudre, Elodie Siband, Bruno Gallas, Fabrice Cousin, Dominique Hourdet, Yvette Tran

**Affiliations:** 1 Ingénierie des Matériaux Polymères, Université Claude Bernard Lyon 1, Université de Lyon, CNRS UMR 5223, 69100 Villeurbanne, France; 2 Soft Matter Sciences and Engineering, ESPCI Paris, PSL Université, Sorbonne Université, CNRS, 10 rue Vauquelin, F-75005 Paris, France; elodiesiband@hotmail.com (E.S.); dominique.hourdet@espci.fr (D.H.); 3 Sorbonne Université, CNRS-UMR 7588, Institut des NanoSciences de Paris, INSP, 4 place Jussieu, F-75005 Paris, France; bruno.gallas@insp.upmc.fr; 4 Laboratoire Léon Brillouin, Université Paris-Saclay, CEA-CNRS, Saclay, 91191 Gif-sur-Yvette CEDEX, France; fabrice.cousin@cea.fr

**Keywords:** responsive brushes, temperature-responsive, pH-responsive, swelling, adsorption, complexation, scaling laws, density profile, neutron reflectivity

## Abstract

We investigate the adsorption of pH- or temperature-responsive polymer systems by ellipsometry and neutron reflectivity. To this end, temperature-responsive poly (*N*-isopropylacrylamide) (PNIPAM) brushes and pH-responsive poly (acrylic acid) (PAA) brushes have been prepared using the “grafting onto” method to investigate the adsorption process of polymers and its reversibility under controlled environment. To that purpose, macromolecular brushes were designed with various chain lengths and a wide range of grafting density. Below the transition temperature (LCST), the characterization of PNIPAM brushes by neutron reflectivity shows that the swelling behavior of brushes is in good agreement with the scaling models before they collapse above the LCST. The reversible adsorption on PNIPAM brushes was carried out with linear copolymers of *N*-isopropylacrylamide and acrylic acid, P(NIPAM-*co*-AA). While these copolymers remain fully soluble in water over the whole range of temperature investigated, a quantitative adsorption driven by solvophobic interactions was shown to proceed only above the LCST of the brush and to be totally reversible upon cooling. Similarly, the pH-responsive adsorption driven by electrostatic interactions on PAA brushes was studied with copolymers of NIPAM and *N*,*N*-dimethylaminopropylmethacrylamide, P(NIPAM-*co*-MADAP). In this case, the adsorption of weak polycations was shown to increase with the ionization of the PAA brush with interactions mainly located in the upper part of the brush at pH 7 and more deeply adsorbed within the brush at pH 9.

## 1. Introduction

Polymer brushes are densely packed assemblies of polymer chains that are end-attached to a surface or interface [1,2]. On planar substrates, they can be obtained with various chemical compositions, polymer chain lengths or grafting densities [3,4]. Water-immersed polymer brushes can be sensitive to changes of temperature [5], pH or ionic strength [6], and other stimuli depending on the nature of the polymer: Upon stimulus, the brushes adapt their conformation. Such change can be used to control the surface properties in order to design smart surfaces exhibiting responsive adsorption [7], specific adhesion [8], tunable wettability [9], or changes in lubrication [10]. As a consequence, responsive polymer brushes have numerous applications, including sensors [11], antifouling surfaces [12], or controlled release [13].

Poly (*N*-isopropylacrylamide) (PNIPAM) exhibits a lower critical solution temperature (LCST) in water and it is certainly among the most popular temperature-responsive polymers [14,15,16,17]. PNIPAM responds to temperature by changing the chains conformation through a coil-to-globule transition around 32 °C, leading to switchable interactions with specific molecules. Responsive to pH, poly (acrylic acid) (PAA) also exhibits a change in conformation. PAA is a neutral coil in acidic pH and it shifts to a stretched conformation when it is ionized in basic conditions. Between its neutral state and its ionized state, PAA can modulate its interactions with other neutral or charged molecules. Both PNIPAM [18,19,20,21] and PAA [13,22,23] are envisioned for applications in the biological field for the control of protein adsorption or cell adhesion.

Polymer brushes on planar substrates can be obtained using different strategies. The physisorption of diblock copolymers containing a surface-anchoring block and the block of interest has the disadvantage of limited applications due to the reversibility of the physical interactions. To covalently end-attach polymer chains, the “grafting from” method—usually based on surface-initiated controlled radical polymerization—is a broadly used method [23,24,25,26,27,28,29]. The “grafting onto” method, which we chose, does not give access to very high grafting densities, but it has the great advantage of simplicity and allows the synthesis of brushes of very well-controlled chain distribution [30,31].

Using covalent grafting approaches, a large number of authors have investigated the controllable swelling of PNIPAM and PAA brushes, which is intrinsically connected to the adsorption ability of these systems. In the case of PNIPAM, Yim et al. have synthesized the brushes by grafting from on gold surfaces and studied the conformational changes by neutron reflectivity [16,32,33,34]. Other studies have reported changes of properties at the coil-to-globule transitions using other experimental techniques [14,24,35]. In the case of PAA, the pH induced transition from the neutral brush to the polyelectrolyte brush has been studied by various groups using neutron reflectivity [36], AFM [6], or spectroscopic ellipsometry [37] in order to determine the different regimes of swelling which depend on pH, ionic strength, and grafting density. Other teams have investigated the dissociation behavior of the PAA brushes, for instance using infrared spectroscopy ellipsometry [38] or a combination of infrared spectroscopy and contact angle titration [39].

In addition to these studies on the swelling properties of brushes, various investigations have been devoted to the responsive adsorption properties of these systems towards polymer [5] or inorganic [40] particles, proteins [22,41,42,43] or cells [18,23]. In this framework, the use of simple model systems should allow a better understanding of the mechanisms that control the molecular interactions involved in the adsorption process. Accordingly, the choice of model interacting (macro) molecules is essential as their solubility can be easily tuned according to their structural parameters.

For this reason, we have designed poly (*N*-isopropylacrylamide-*co*-acrylic acid) (P(NIPAM-*co*-AA)) and poly (*N*-isopropylacrylamide-*co*-*N*, *N*-dimethylaminopropylmethacryl amide) (P(NIPAM-*co*-MADAP)) copolymers with NIPAM:AA and NIPAM:MADAP molar ratios equal to 9:1 [44]. While NIPAM forms temperature-responsive polymers, AA units are neutral at low pH and anionic at high pH (pK_a_ ~ 4.5) and MADAP units are cationic at low pH and neutral at high pH (pK_a_ ~8.2). These two copolymers are model macromolecules whose hydrophobicity and ionicity can be easily tuned with pH and temperature [45]. For these copolymers, when the ionizable monomers are neutral (low pH for AA and high pH for MADAP), the copolymers undergo a temperature-responsive phase separation in the temperature range 20–60 °C. On the contrary, when AA or MADAP are ionized, no transition can be observed in the same temperature range. In this article, we investigate the adsorption behavior of such copolymers on temperature-responsive brushes of PNIPAM at pH 7 and on pH-responsive PAA brushes at ambient temperature. To this end, a careful analysis of the swelling behavior of the PAA brushes (published elsewhere [36]) and PNIPAM brushes was carried out by ellipsometry and neutron reflectivity. The combination of these techniques gives access to the amount of adsorbed polymer depending on the chain lengths and on the grafting densities of the PAA and PNIPAM brushes.

## 2. Materials and Methods

### 2.1. Synthesis of PNIPAM and PAA Brushes

The synthesis of the PAA and PNIPAM brushes followed steps reported elsewhere [31]. After being cleaned and rejuvenated by immersion in freshly prepared “piranha” solution (70 vol % of sulfuric acid (97%) and 30 vol % of hydrogen peroxide (35%) heated at 150 °C for 20 min), the silicon substrates (380 µm thick wafers from ACM, or monocrystals of particular size (100 × 50 × 10 mm^3^) adapted for neutron reflectivity) were then rinsed with pure water (Millipore, resistivity ≥ 18.2 MΩ cm). They were also cleaned by ultrasound in water for 1 min and finally dried with a nitrogen flow.

Then, an epoxy-functionalized silane self-assembled monolayer was formed by exposing the freshly cleaned silicon wafers to a 2 vol % 3-glycidoxypropyltrimethoxysilane (GPS, Gelest, Inc., 97%, Morrisville, PA, USA) solution in anhydrous toluene (Aldrich, 99.8%, Saint Louis, MO, USA) for 5 h.

For the synthesis of PNIPAM brushes, carboxy-terminated poly (*N*-isopropylacrylamide) (PNIPAM-COOH, from Polymer Source) of three different molar masses (*M*_n_ = 121 kg/mol, *Ð* = 2.5; *M*_n_ = 70 kg/mol, *Ð* = 2.3 and *M*_n_ = 11.9 kg/mol, *Ð* = 2.2) were grafted by ring-opening reaction of PNIPAM-COOH with surface-attached GPS molecules. A PNIPAM-COOH film was spread on the GPS monolayer by spin-coating from 1 wt % tetrahydrofuran (THF, Carlo Erba Reagents, 95%, Val de Reuil, France) solution. The samples were heated at 150 °C in a vacuum oven. The polymer solutions for spin-coating contained mixtures of PNIPAM-COOH functionalized chains and PNIPAM passive chains with various ratios in order to obtain PNIPAM brushes with different grafting densities. Various reaction times of annealing were also tested. The silicon wafers were then rinsed extensively with THF to remove the ungrafted polymer chains. The substrates were finally sonicated in THF for 1 min and dried with a nitrogen flow.

For the synthesis of PAA brushes, carboxy-terminated poly (*tert*-butyl acrylate) (PtBuA-COOH, from Polymer Source) with various molar masses (*M*_n_ = 42 kg mol^−1^, *Ð* = 1.12; *M*_n_ = 6.5 kg mol^−1^, *Ð* = 1.08 and *M*_n_ = 4.2 kg mol^−1^, *Ð* = 1.25) were grafted by ring-opening reaction of PtBuA-COOH with surface-attached GPS molecules. A PtBuA reservoir was spread from 1 wt % THF solution and some of the chains were grafted by the ring-opening reaction of the carboxy end-group of the PtBuA-COOH with the surface-attached GPS by heating at 120 °C under vacuum for 24 h. The ungrafted chains were then removed by extensive rinsing of the wafers in THF and sonication in THF for 2 min. The substrates were finally dried under a nitrogen stream. This grafting process was performed first for “long” chains (*M*_n_ = 42 kg mol^−1^) and then for “very short” chains (*M*_n_ = 4.2 kg mol^−1^). This added layer of very short chains allows the cover of the GPS silane molecules that are not connected to PtBuA long chains. Finally, the PtBuA brushes were converted into PAA brushes by pyrolysing the PtBuA-functionalized silicon wafers for 2 h at 200 °C under vacuum, then by immersing the brushes in water at pH 2 overnight and by rinsing and drying the substrates with a nitrogen flow.

The characteristics of PNIPAM and PAA brushes synthesized are displayed in Table 1.

### 2.2. Synthesis of Amino-Terminated P(NIPAM-co-AA) and P(NIPAM-co-MADAP) Telomers

#### 2.2.1. Synthesis of Amino-Terminated P(NIPAM-*co*-MADAP)

The synthesis of copolymers containing *N*-isopropylacrylamide and *N*-[3-(dimethylamino) propyl]-methacrylamide (MADAP) was achieved by telomerisation to control the end group of the polymer, its molar mass, and its composition [44,45]. The synthesis can be summarized as follows: 90 mmol of monomers (81 mmol for NIPAM and 9 mmol for MADAP) were dissolved in 100 mL of water and the solution was deoxygenated during 1 h under nitrogen bubbling. Potassium persulfate KPS (0.9 mmol) and 2-aminoethane thiol hydrochloride AET, HCl (1.8 mmol) as redox initiators were separately dissolved in 10 mL of water before addition to the solution of monomers. The reaction was allowed to proceed at 20 °C to avoid phase separation (PNIPAM LCST is around 32 °C). An appropriate amount of sodium hydroxide was added after 4 h to neutralize HCl. The polymer was then purified by dialysis against pure water (membrane cut-off = 6–8 kDa) and recovered by freeze-drying. The reaction yield was between 65–85 wt %. The composition (NIPAM:MADAP 9:1) and molar masses of the copolymers were obtained by SEC, titration, and ^1^H NMR.

#### 2.2.2. Synthesis of Amino-Terminated P(NIPAM-*co*-AA)

The synthesis of amino-terminated copolymers containing *N*-isopropylacrylamide (NIPAM, 97%, Aldrich, Saint Louis, MO, USA) and acrylic acid (AA) was achieved by telomerization and can be summarized as follows: 9 g (80 mmol) of NIPAM and 0.64 g (9 mmol) of AA were dissolved in 100 mL of water and the solution was deoxygenated during 1 h under nitrogen bubbling. Sodium persulfate KPS (0.9 mmol) and AET, HCl (1.8 mmol) as redox initiators were separately dissolved in 10 mL of water before addition to the NIPAM solution. The reaction was allowed to proceed at 20 °C to avoid the phase separation of the polymer. An appropriate amount of sodium hydroxide was added after 4 h to neutralize the hydrochloride ions and the acrylic acid. The polymer was then purified by dialysis against pure water (membrane cut-off = 6–8 kDa) and recovered by freeze-drying. The reaction yield was between 70 and 80 wt %. The composition and molar mass of the copolymers were obtained by SEC, titration, and ^1^H NMR. The characteristics of the P(NIPAM-*co*-AA) copolymer obtained by SEC are: *M*_n_ = 18.8 kg mol^−1^ and *Ð* = 1.4. The ratio of AA is comparable to what was expected: 7% by titration and 10% by ^1^H NMR, the value obtained by titration could be under-estimated due to the titration of amino end-groups.

### 2.3. Ellipsometry

Ellipsometry measurements were performed on a spectroscopic apparatus from SOPRA (ES4G). The wavelength ranged from 300 to 750 nm and the angle of incidence was set to 70°. Both in-air and in-solution measurements were performed, the latter by using a liquid cell with thin glass walls fixed perpendicularly to the light path. A multilayer model for a flat film was used for the calculation of the thickness of silica, initiator, and grafted polymer layers from the experimentally measured ellipsometric angular functions tan Ψ and cos Δ. The refractive indices *n* used for the calculations were 3.874 for the silicon substrate, 1.460 for the native silica layer. We also used *n* = 1.460 for the GPS self-assembled monolayer, *n* = 1.520 for the PNIPAM dry brushes, and *n* = 1.527 for the PAA dry brushes. Using the software WinElli, both refractive index and thickness of the swollen polymer layer were extracted from the best fit of the ellipsometric data.

### 2.4. Neutron Reflectivity

Neutron reflectivity measurements were performed at silicon–liquid interface on the reflectometer EROS at the Laboratoire Léon Brillouin (CEA, Saclay, France). The experimental procedure and setup were described in detail in previous publications [36,46]. Neutron reflectivity experiments were carried out with protonated polymer brushes (without and with protonated free copolymers) and deuterated water in order to determine the monomer density profile of the brushes (without and with the adsorbed copolymers). The experimental setup is usual for silicon–liquid interface studies. The sample holder maintains the 100 × 50 × 10 mm^3^ silicon block tightly clamped against a Teflon trough filled with the liquid solution. The neutron beam crosses through the silicon crystal before reflecting at the silicon–liquid interface. Reflectivity was measured at the incident angle of 1.34° with neutrons of wavelength ranging from 3 to 22 Å.

The neutron reflectivity is sensitive to the profile of the scattering length density in the direction normal to the interface *Nb*(*z*). A reliable model-independent method was chosen to determine *Nb*(*z*). The brush was modeled as a set of layers, each characterized by a fixed thickness and a fixed scattering length density. Two adjacent layers were connected using error functions (erf) of fixed width to get a continuous profile. The procedure consisted of choosing a profile of scattering length density and finding the corresponding parameters giving the best fitting of the experimental reflectivity data. This reliable method allowed the determination of a continuous profile of scattering length density without making any assumption about its analytical form.

The monomer volume fraction profile *ϕ*(*z*) was deduced from *Nb*(*z*) using the relation:

(1) $$\phi \left(z\right)=\frac{Nb\left(z\right)\;-\;Nb_{\mathrm{polymer}}}{Nb_{\mathrm{solvent}}\;-\;Nb_{\mathrm{polymer}}}$$

where *Nb*_polymer_ and *Nb*_solvent_ are respectively the scattering length densities of the polymer and solvent. The scattering length densities used for the calculation are: 6.40 × 10^−6^ Å^−2^ for heavy water, 1.36 × 10^−6^ Å^−2^ for PNIPAM. As the concentration of P(NIPAM-*co*-AA) copolymers and PAM-*g*-P(NIPAM-*co*-AA) comb-polymers in the aqueous solution is low (10 g L^−1^ or about 1% *v*/*v* at the maximum), the presence of the polymer does not change the scattering length density of the liquid phase as shown by the same position of the critical wave vector. For the samples with the single PNIPAM brushes, we used *Nb* = 1.36 × 10^−6^ Å^−2^. For the samples with the adsorbed linear polymer and comb-copolymer, we also used *Nb* = 1.36 × 10^−6^ Å^−2^ for two main reasons. First, the scattering length densities of PAM and PNIPAM are very close and the PAM-*g*-P(NIPAM-*co*-AA) contains a majority of acrylamide (6900 AM units compared to 19 P(NIPAM-*co*-AA) grafts). Second, the P(NIPAM-*co*-AA) copolymers which contain only 10 mol % of AA (*Nb*_PAA_ = 2.75 × 10^−6^ Å^−2^) should have *Nb* = 1.49 × 10^−6^ Å^−2^. Nevertheless, assuming that the whole adsorbed layer including the PNIPAM brush and the P(NIPAM-*co*-AA) copolymer has *Nb* = 1.36 × 10^−6^ Å^−2^ (instead of *Nb* = 1.49 × 10^−6^ Å^−2^) provided less than 5% of uncertainty for the density profile *ϕ*(*z*) (and also for the dry thickness *γ* or the swollen thickness *L*). All the scattering length densities had to be subtracted from the scattering length density of silicon (equal to 2.07 × 10^−6^ Å^−2^) since in the experimental setup, the incoming neutron beam passes through the silicon block.

From the density profile *ϕ*(*z*), we can calculate the dry thickness *γ* of the polymer layer, equal to the zero-th order moment of *ϕ*(*z*) (or the integral of the profile):

(2) $$\gamma =\int_{0}^{+\infty }\phi \left(z\right)dz$$

The dry thickness *γ* is an important parameter because it is independent of the shape of *ϕ*(*z*). It corresponds to the thickness of the dry layer or also the amount of polymer per unit area. It had to be compared with the values measured by other techniques such as ellipsometry. This parameter was used consequently to validate the density profile obtained by neutron reflectivity. For the measurements of the polymer brush in water, *γ* should correspond to the dry thickness of the single brush. If the adsorption measurements are achieved with the polymer brush in contact with aqueous solutions containing linear polymers or comb-polymers, *γ*_total_ corresponds to the dry thickness of the whole layer including the polymer brush and the adsorbed polymers. Knowing the dry thickness of the brush, the dry thickness of the adsorbed layer *γ*_ads_ which also corresponds to the amount of adsorbed polymers can be calculated.

The swollen thickness *L* can also be deduced from the profile. It is proportional to the normalized first order moment of *ϕ*(*z*):

(3) $$L=2\frac{\int_{0}^{+\infty }z\phi \left(z\right)dz}{\int_{0}^{+\infty }\phi \left(z\right)dz}$$

The swollen thickness is twice the normalized first moment of the volume fraction profile which is defined with complementary error functions, erf, connecting two adjacent layers in order to get a decaying profile. As for the dry thickness *γ*, the swollen thickness *L* can be associated either to the single polymer brush in water or to the whole adsorbed layer including the polymer brush and the adsorbed polymers.

## 3. Results and Discussion

### 3.1. Characterization of Dry PNIPAM Brushes

The dry thicknesses of PNIPAM brushes *γ* were characterized by ellipsometry. From these measurements, we deduced the grafting densities *σ* and the average distance between anchoring sites *D*. The characteristics of PNIPAM brushes are indicated in Table 1. For brushes with the same molar mass, the variation of the grafting density was obtained either by using mixtures of carboxy-terminated chains and non-functionalized chains with different ratios (10% and 100%) or with different reaction times. The brushes with the lowest densities are unsurprisingly those synthesized with the lowest ratio of functionalized chains and the shortest reaction time. The comparison of the distance *D* with the size of the chain in Θ-conditions (2 *R*_0_) demonstrates that all the PNIPAM samples are definitely in the brush regime as the ratio 2 *R*_0_/*D* = 2–5 and should be even larger in good solvent conditions (further discussion available in Supporting Information). The situation is different in the collapsed state where the average distance between anchored chains becomes similar to the size of the dry PNIPAM globule, even smaller for half of the samples: *bN*^1/3^/*D* = 0.6–1.5 (0.7–1.9 if we consider the presence of 50% of water). Therefore, we can conclude that in the collapsed state the level of confinement is much weaker for PNIPAM globules which cannot be truly considered in the “brush regime”. The coil–globule transition of the PNIPAM chain observed when crossing the LCST is then expected to induce a shift from the semi-dilute polymer brush regime to the mushroom regime for PNIPAM layers of low grafting density which will not be considered in the following. From this first set of experiments, we can conclude that the grafting onto the procedure is very efficient to prepare, from well-controlled molar masses (from 12 to 120 kg mol^−1^), polymer brushes with a large range of grafting densities (*σ* = 0.015 to 0.22 nm^−2^).

### 3.2. Swelling Behavior of PNIPAM Brushes below LCST

Neutron reflectivity was used to study PNIPAM brushes in water. Figure S1 shows an example of neutron reflectivity data and the profile of the volume fraction of monomers corresponding to the best fit of the experimental results. The analytical forms of the density profile of polymer brushes were investigated in detail in previous publications and in particular for poly (acrylic acid) brushes [36]. In the present work, we aim at comparing the density profiles of polymer brushes at different temperatures, with or without adsorbed polymers, a simple step model is well adapted.

**Figure 1:**
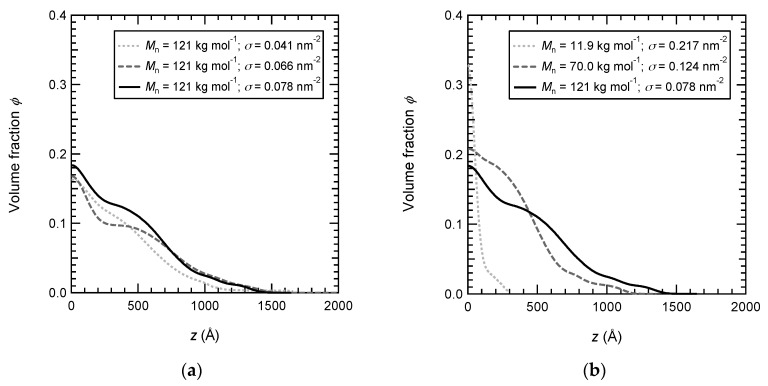
Polymer density profiles of PNIPAM brushes in D2O at 20 °C. (**a**) PNIPAM brushes have the same molar mass (*M*_n_ = 121 kg·mol^−1^) and various grafting densities: 0.078 nm^−2^ (black and solid line), 0.066 nm^−2^ (grey and dashed line), and 0.041 nm^−2^ (grey and dotted line). (**b**) PNIPAM brushes have various molar masses and various grafting densities (the densest brush being chosen): 121 kg·mol^−1^ and 0.078 nm^−2^ (black and solid line), 70 kg·mol^−1^ and 0.124 nm^−2^ (black and dotted line), and 11.9 kg·mol^−1^ and 0.217 nm^−2^ (black and dotted line).

The volume fraction profiles of PNIPAM brushes with various grafting densities and molar masses are displayed in Figure 1. For the series with the same molar mass and various grafting densities, the density profiles are rather comparable since the range investigated is very tiny (with a factor 2 between 0.041 and 0.078 nm^−2^). The surface-attached chains can extend up to 1500 Å (at the maximum) from the surface. Conversely, the density profiles shown in Figure 1b become much more different when the molar mass of brush chains is varied over a wide range (from *M*_n_ = 11.9 to 121 kg mol^−1^). The PNIPAM brush with short chains is much more localized in the vicinity of the surface and its extension is limited to a distance of 200 Å from the surface. The volume fraction at the surface is consistently the highest (but below 0.35) compared to that of the two other brushes since the brush is very dense with a grafting density equal to 0.217 nm^−2^.

From the volume fraction profiles, the average thickness of the swollen brush *L* is deduced from Equation (3). The swelling ratio, defined as the ratio of the thickness of the swollen brush to that of the dry brush, *L*/*γ*, allows quantifying the stretching of the chains in solvent and it follows a scaling relation (see SI):

(4) $$\frac{L}{\gamma }\propto N^{0}\sigma^{\beta },$$

with *β* = −2/3, −1/2, and 0 for good, Θ- and poor solvents, respectively.

From Equation (4) the swelling ratio of polymer brushes should be independent of the molar mass, whatever is the solvent quality. The data are given in Table 2, and the swelling ratio have been plotted (see Figure S2); the best fit of the data gives a power law with a scaling exponent −0.69 which is in good agreement with the theoretical value predicted for semi-dilute polymer brushes in good solvent, as this is the case for PNIPAM at 20 °C.

### 3.3. Shrinkage of PNIPAM Brushes above LCST

In the present work, we chose to study PNIPAM at only two temperatures, 20 and 60 °C, which are respectively below and far above the LCST of PNIPAM brushes (around 30 °C) to be sure to really investigate PNIPAM chains in their swollen and collapsed states. Indeed, it has been shown by Bittrich et al. [30], that the temperature range of the swollen-collapsed transition of PNIPAM brushes could be broadened (between 22 and 32 °C) with decreasing grafting density (in the range 0.04 to 0.11 nm^−2^).

**Figure 2:**
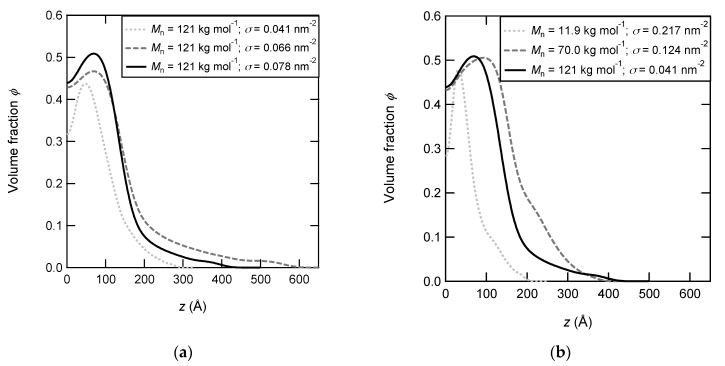
Polymer density profiles of PNIPAM brushes in D2O at 60 °C. (**a**) PNIPAM brushes have the same molar mass (*M*_n_ = 121 kg mol^−1^) and various grafting densities: 0.078 nm^−2^ (black and solid line), 0.066 nm^−2^ (grey and dashed line), and 0.041 nm^−2^ (grey and dotted line). (**b**) PNIPAM brushes have various molar masses (and various grafting densities, the densest brush being chosen): 121 kg mol^−1^ and 0.078 nm^−2^ (black and solid line), 70 kg mol^−1^ and 0.124 nm^−2^ (black and dotted line), and 11.9 kg mol^−1^ and 0.217 nm^−2^ (black and dotted line).

The volume fraction profiles of PNIPAM brushes at 60 °C are displayed in Figure 2. For all the samples, the profiles show the collapse of PNIPAM brushes at 60 °C if compared to those obtained at 20 °C in Figure 1.

For the series prepared with the same molar mass and various grafting density, the three density profiles are unsurprisingly similar at 60 °C as they were at 20 °C. The slight difference is only due to the variation of the dry thickness or the integral of the profile. Obviously, the volume fraction in the polymer brush is much higher at high temperature. For the series with various molar masses (Figure 2b), if the density profiles were rather dissimilar at 20 °C, the difference between the profiles is greatly reduced at 60 °C. Indeed, all the collapsed profiles become comparable at 60 °C for short chains as well as long chains (except the small variation of the integral of the profile). The highest volume fraction is about 0.5 and all brushes with long chains (*M*_n_ = 70 and 121 kg mol^−1^) extend at only 300 Å from the surface, compared to 1500 Å at 20 °C. All the profiles show a depletion layer which is quite deep over the first 50 Å for the brush with short chains (*M*_n_ = 11.9 kg mol^−1^) and less pronounced but broader for brushes with long chains (*M*_n_ = 121 kg mol^−1^ and *σ* = 0.041 nm^−2^). The depletion layer is a little bit thicker for denser brushes or brushes with long chains. Our profiles with the depletion layer differ from those reported by Yim et al. [32]. They found a bilayer profile composed of a very thin layer of high concentration near the surface followed by a second layer of very low concentration. They explained the shape of the profile by hydrophobic interactions of PNIPAM chains with the surface functionalized by a methyl-terminated self-assembled monolayer (used for the synthesis by “grafting onto”). In our case, the PNIPAM brushes were also synthesized using the “grafting onto” strategy but with a hydrophilic GPS self-assembled monolayer terminated with OH groups. Above the LCST, the hydrophobic interactions between the isopropyl groups of NIPAM units leading to the collapse of PNIPAM brushes are predominant and overcome the H-bonds interaction with hydroxyl-terminated surface. Moreover, we can also assume that the hydration level remains higher close to this hydrophilic surface.

From the calculation of the average thickness of collapsed brushes (*L*_60_) given in Table 2, one can have a quantitative picture of the swollen/collapsed transition undergone by PNIPAM layers.

First, we can see that the average size of individual chains inside the brush, (*L*_60_*D*^2^)^1/3^ is highly reduced at 60 °C but remains higher than the distance between two grafted chains. That means that the brush regime is retained in the collapsed state for these samples of highest grafting density. The one-dimension collapse of PNIPAM brushes between 20 and 60 °C can be estimated by the ratio between the layer thicknesses determined at these two temperatures (L_20_/L_60_). If we consider that in the collapsed state the PNIPAM globule is dried, we should have a swollen to collapsed ratio (L_20_/L_60_) identical to the former swelling ratio calculated from the thickness of the swollen layer divided by its thickness in the dry state (L_20_/*γ*). As shown in Figure 2 this is clearly not the case as PNIPAM brushes retain water even at 60 °C when polymer chains are in the globular state. This is a quite general result which has been reported by a large number of authors [30,34,47].

**Figure 3:**
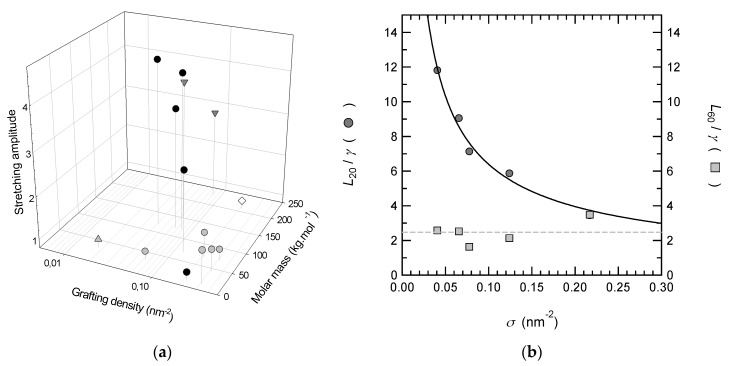
(**a**) Swollen/collapsed ratio of PNIPAM brushes as a function of the grafting density and the molar mass. It corresponds to the ratio of the swollen thickness (for temperatures below the LCST) to the collapsed thickness (for temperatures above the LCST). Our results (●) are compared to those obtained by Yim et al.: (●) [34], (▼) [33], and (▲) [32]. (**b**) Swelling ratio measured at 20 (*L*_20_/*γ*) and at 60 °C (*L*_60_/*γ*) of the immersed PNIPAM brushes as a function of the grafting density; while *L*_60_/*γ* is somewhat constant, *L*_20_/*γ* can be fitted with the following power law: 1.2 × σ^−0.72^.

In order to compare our results with those of Yim et al. [33] who have carried out similar experiments with PNIPAM brushes prepared by ATRP on gold or silica surfaces, we have plotted in Figure 3a the variation of the swollen/collapsed ratio as a function of the grafting density and the molar mass. This compilation of data, performed over a broad range of grafting density (from 0.01 to 0.54 nm^−2^) and molar masses (from 12 to 230 kg mol^−1^), offers a large overview of the swelling behavior of PNIPAM brushes which does not seem to depend from the way of synthesis (grafting from or grafting onto). From this representation the two main tendencies are that the swollen/collapsed ratio remains rather weak when the molar mass is low or when the grafting density is high. Indeed, the deswelling ratio is very weak for molar masses below 50 kg mol^−1^ over the entire range of grafting density. It can even be close to 1 (no deswelling) as we have seen with the brush prepared with 12 kg mol^−1^ PNIPAM chains. Experimental swelling ratios obtained with polymer brushes of different molar masses have been plotted in Figure 3b as a function of the grafting density. The best fit of the data gives a power law with a scaling exponent −0.72 which is in good agreement with the theoretical value predicted for semi-dilute polymer brushes in good solvent, as this is the case for PNIPAM at 20 °C. The pre-factor 1.2 is indicative that the hydrated monomer size is about 20% larger than the dry monomer size. This behavior is also in good agreement with the work of Zhu et al. [17] who shows that PNIPAM brushes, prepared with low molar masses (2.5–10 kg mol^−1^), remain swollen and do not collapse above the LCST. Plunkett et al. [15] also demonstrated that the collapse of chains above the LCST is less pronounced for brushes with low molar mass at low grafting density. These results are also in good agreement with numerical simulations performed by Mendez et al. [48] in the case of PNIPAM brushes; they have shown that the maximum deswelling ratio should be reached at intermediate grafting densities. They also predict that the deswelling ratio increases with the molar mass of polymer chains with a maximum expected at lower grafting density when the molar mass increases.

Finally, if the responsiveness of the polymer brush is the key parameter, PNIPAM brushes with high molar mass (more than 100 kg mol^−1^) and intermediate grafting density (about 0.1 nm^−2^) will be the most efficient.

### 3.4. Complexation of PNIPAM Brushes with P(NIPAM-co-AA) Linear Copolymers

The adsorption of P(NIPAM-*co*-AA) linear copolymers on PNIPAM brushes was investigated by neutron reflectivity at solid–liquid interface. The solid substrate was silicon wafer with surface-attached PNIPAM brushes. The liquid phase was an aqueous solution of P(NIPAM-*co*-AA) copolymer with a concentration of 1 g L^−1^ at pH 7 to avoid the phase separation of added chains. Two other concentrations 0.5 and 10 g L^−1^ were also studied with the same results. pH 7 was chosen to avoid the formation of aggregates. It was shown in a previous paper that P(NIPAM-*co*-AA) are not soluble for pH below 5 at high temperature (in particular at 60 °C) whereas they are soluble in water at room temperature for any pH. Indeed, the copolymers are soluble at any temperature for pH above 7. It means that the complexation of P(NIPAM-*co*-AA) copolymers and PNIPAM brushes with temperature should be ideally studied at pH 7 (or higher pH) as there is no effect of pH on the adsorption.

The adsorption of P(NIPAM-*co*-AA) linear copolymers on PNIPAM brushes was found to be reversible with temperature. At room temperature, there was no adsorption. At 20 °C, the reflectivity curves and accordingly the density profiles of the brush in P(NIPAM-*co*-AA) aqueous solution were the same as those in water.

**Figure 4:**
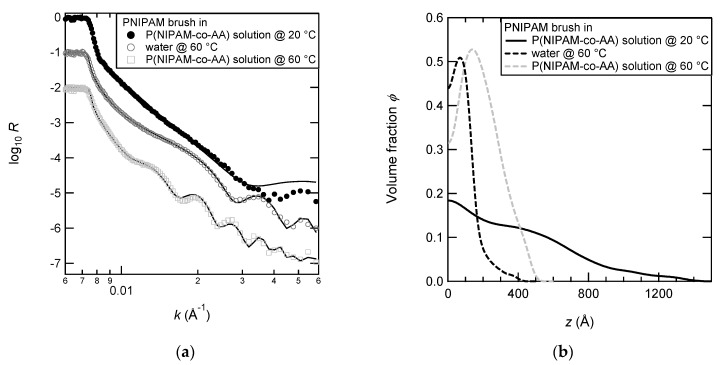
Neutron reflectivity data (**a**), markers, and profiles of volume fraction of monomers (**b**) corresponding to the best fit of the reflectivity data (**a**), solid lines. The samples investigated are: PNIPAM brush at 20 °C (filled circles on the left; grey and solid line on the right), PNIPAM brush at 60 °C (open circles on the left; black and dashed line), and PNIPAM brush at 60 °C with the adsorbed P(NIPAM-*co*-AA) linear copolymer (crosses on the left; black and dotted line on the right). The PNIPAM brush has these following characteristics: *M*_n_ = 121 kg mol^−1^, *γ* = 113 Å, and *σ* = 7.76 × 10^−2^ nm^−2^.

Figure 4 shows the neutron reflectivity data and the density profiles (corresponding to the best fit of the experimental data) of PNIPAM brush at 60 °C in D_2_O solution containing 1 g L^−1^ of P(NIPAM-*co*-AA) copolymers in comparison with those in pure water at 20 and 60 °C. The reflectivity curves of the PNIPAM brush at 60 °C with and without adsorbed P(NIPAM-*co*-AA) display more obvious Kiessig fringes than the curve at 20 °C. The corresponding density profiles are consistently less smooth than the profile at 20 °C. The PNIPAM brush at 60 °C with and without adsorbed P(NIPAM-*co*-AA) is less extended from the surface than the brush at 20 °C. Both density profiles at 60 °C show a slight depletion layer (already discussed in the previous part). The profile of the brush in the copolymer solution is much broadened than that in pure water at 60 °C indicating the presence of the adsorbed copolymer in the additional part of the profile. It definitely demonstrates that P(NIPAM-*co*-AA) free copolymers adsorb on top of the collapsed PNIPAM brush. When returning to 20 °C, the reflectivity measured is the same to the one obtained prior to the experiment at 60 °C, demonstrating the reversibility of the adsorption.

**Figure 5:**
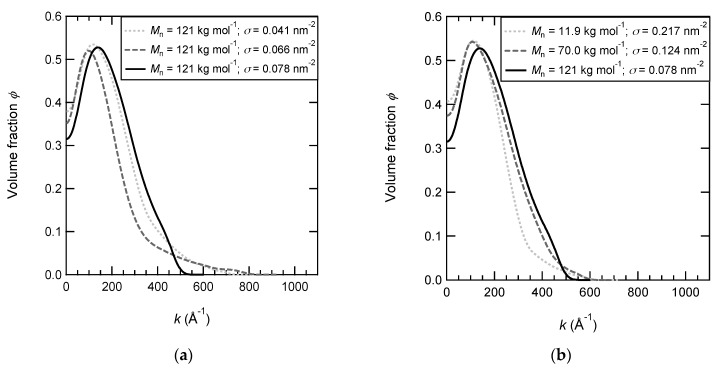
Polymer density profiles of PNIPAM brushes at 60 °C with the adsorbed P(NIPAM-*co*-AA) linear copolymers. (**a**) PNIPAM brushes have same molar mass (*M*_n_ = 121 kg mol^−1^) and various grafting density: 0.078 nm^−2^ (black and solid line), 0.066 nm^−2^ (grey and dashed line), and 0.041 nm^−2^ (grey and dotted line). (**b**) PNIPAM brushes have various molar mass: 121 kg mol^−1^ (black and solid line), 70 kg mol^−1^ (black and dotted line), and 11.9 kg mol^−1^ (black and dotted line).

Figure 5 displays the volume fraction profiles of PNIPAM brushes of various grafting density and various molar mass in the presence of adsorbed P(NIPAM-*co*-AA). The profiles are all rather similar. The comparison with the profiles of the collapsed PNIPAM brushes at 60 °C (see Figure 4) shows that the adsorption is a little higher for the sparsest brush with long chains. It could be explained by an easier interpenetration of P(NIPAM-*co*-AA) free chains into PNIPAM brush which helps the complexation of chains. The adsorption is also higher for the brush with short chains, probably due to a better accessibility to the PNIPAM brush. For all samples, the profile of the brush in the copolymer solution is more extended than that in pure water with a broadened region averaging 200 Å. It indicates that the adsorbed copolymer is localized in the additional part of the profile. Again, it confirms that the adsorption occurs on the top of the collapsed brush. The adsorption is indeed governed by hydrophobic interactions between the P(NIPAM-*co*-AA) free chains and the PNIPAM surface-attached chains. The modes of interaction are likely secondary and/or ternary adsorption, but absolutely not primary adsorption. As described by Currie et al. [49] the adsorption can take place at the brush–water interface (coined secondary adsorption), it can also be either within the grafted layer or on the grafted chains (coined ternary adsorption). In the primary mode, the adsorption occurs at the grafting surface by way of the diffusion of the adsorbed particle through the brush. In our case, the copolymer (*M*_n_ = 18.8 kg mol^−1^) is unable to diffuse through the collapsed and dense brush (the distance between two surface-attached chains is 50 Å at the maximum). Actually, P(NIPAM-*co*-AA) free chains adsorb mainly on the top of the collapsed PNIPAM brushes.

From the density profiles, some quantitative analyses on the adsorption were achieved as indicated in Table 3. The dry thickness *γ*_total_ and the swollen thickness *L*_total_ of the whole layer including the PNIPAM brush and the adsorbed P(NIPAM-*co*-AA) copolymer were determined. The dry thickness of the adsorbed layer *γ*_ads_, which is also the amount of adsorbed copolymer per unit area, can be deduced: *γ*_ads_ = *γ*_total_ − *γ* (where *γ* is the dry thickness of the brush). The swollen thickness of the adsorbed layer *L*_ads_ can also be calculated: *L*_ads_ = *L*_total_ − *L* (where *L* is the dry thickness of the brush) if the density profile of the brush is supposed to be unmodified in the presence of the adsorbed copolymer. Moreover, the ratio of the adsorbed amount can be extracted:

(5) $$R_{\mathrm{ads}}=\frac{\gamma_{\mathrm{ads}}}{\gamma }\times \frac{\rho_{\mathrm{copo}}}{\rho_{\mathrm{brush}}}\times \frac{M_{\mathrm{brush}}}{M_{\mathrm{copo}}}$$

where *ρ*_copo_ and *ρ*_brush_ are the density of P(NIPAM-*co*-AA) (*ρ*_copo_ = 1.355 g cm^−3^) and PNIPAM (*ρ*_brush_ = 1.386 g cm^−3^), *M*_copo_ and *M*_brush_ the average molar mass of monomers in P(NIPAM-*co*-AA) chains (*M*_copo_ = 109 g mol^−1^) and in PNIPAM chains (*M*_brush_ = 113 g mol^−1^). The values of *γ*_ads_ and *L*_ads_ are roughly comparable for all the brushes with long chains (*M*_n_ = 121 kg mol^−1^ and 70 kg mol^−1^). For these brushes, the ratios *R*_ads_ are equal to 0.39 or 0.49 for the densest brushes indicating that the amount of adsorbed chains is about half the amount of surface-attached chains. For the sparsest brush, the value of *γ*_ads_ is higher than *γ* and much more higher compared to the other samples. The adsorbed ratio *R*_ads_ is equal to 1.70. It means that the adsorption is much more important for brushes with weak grafting density: The free volume led by the sparse surface-attached chains facilitates the complexation with adsorbed chains. For the brush with short chains (*M*_n_ = 11.9 kg mol^−1^), the adsorption is expected to be weak as the grafting density is high. The adsorption is conversely much better with the highest adsorbed ratio equal to 3.49. Indeed, this brush is not collapsed at 60 °C. Compared to the other collapsed brushes, the brush with short chains shows more monomer units easily accessible for complexation and adsorption. As a result, the adsorption on PNIPAM brushes is promoted for weakly dense brushes and brushes with short chains. In the first case, sparse brushes help the interpenetration and complexation with free chains. In the latter case, brushes with short chains (even dense) which remain rather swollen, even above the LCST, provide the ease of access and complexation for free chains.

Compared to the adsorption of proteins on PNIPAM brushes, the adsorption of P(NIPAM-*co*-AA) linear copolymers is much higher. The lowest amount of adsorbed copolymers we obtained is about 5 mg m^−2^ (corresponding to *γ*_ads_ = 37 Å with *ρ*_copo_ = 1.355 g cm^−3^). Xue et al. [41] found very low levels of BSA (Bovin Serum Albumin) proteins adsorption, the maximum being equal to 0.55 mg m^−2^. They also showed that the amount of adsorbed proteins increases with decreasing grafting densities from 0.3 (for σ = 0.11 nm^−2^) to 0.55 mg m^−2^ (for σ = 0.08 nm^−2^), which is in good agreement with our results. If copolymers adsorb more on PNIPAM brushes than proteins, it is in part due to the linear structure of chains which allows ease of access and complexation. Moreover, Burkert et al. [47] showed that effect of pH on the adsorption of HSA (Human Serum Albumin) proteins, which is chemically equal to BSA proteins, is more important than the effect of temperature. They found that HSA proteins adsorbed more on P2VP brushes (around 7.2 mg m^−2^) than PNIPAM brushes (around 1 mg m^−2^). They concluded that the adsorption of HSA proteins is probably governed by electrostatic interactions rather than hydrophobic forces.

### 3.5. pH-Reversible Adsorption on PAA Brushes

The adsorption of P(NIPAM-*co*-MADAP) copolymers on PAA brushes was investigated using ellipsometry. The measurements were carried out on dry samples and in situ at the solid–liquid interface.

**Figure 6:**
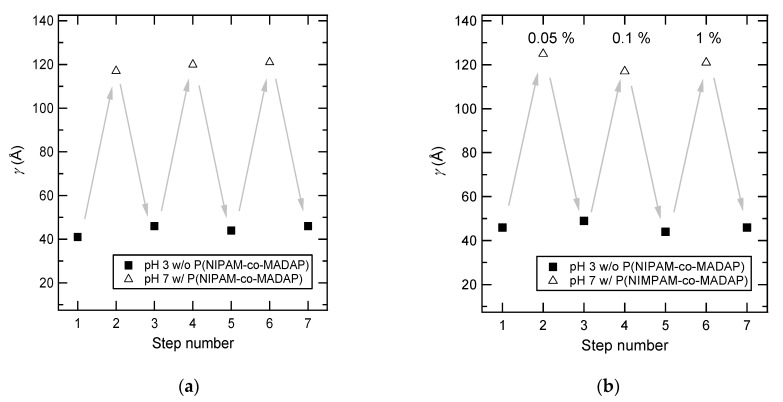
(**a**) Dry thicknesses after desorption in water at pH 3 (*γ*_brush_: ■) and after immersion and adsorption in a 0.1 wt % poly (*N*-isopropylacrylamide-*co*-*N*, *N*-dimethylaminopropylmethacryl amide) (P(NIPAM-*co*-MADAP)) aqueous solution equilibrated at pH 7 (*γ*_tot_: Δ). The desorption/adsorption cycle is repeated three times. (**b**) Dry thicknesses after desorption in water at pH 3 (*γ*_brush_: ■) and after immersion and adsorption in a P(NIPAM-*co*-MADAP) aqueous solution equilibrated at pH 7 (*γ*_tot_: Δ). The desorption/adsorption cycle is carried out three times with varying the P(NIPAM-*co*-MADAP) concentration (cycle 1: 0.05, cycle 2: 0.1, and cycle 3: 1 wt %).

For the measurements on dry samples, the thickness was measured at different steps: (a) The brush was immersed with water at pH 3 and dried (measurement of the PAA brush thickness *γ*_brush_), and (b) the brush was immersed in the copolymer solution at pH 7, rinsed with water at pH 7, and dried (measurement of the thickness of the (brush + adsorbed chains) system *γ*_tot_). This process was repeated several times. The results are displayed in Figure 6. They clearly demonstrate the reversibility of the adsorption. The dry thickness of the adsorbed layer, *γ*_ads_ which is also the amount of adsorbed copolymer per unit area, is the difference between the total thickness with the adsorbed layer *γ*_total_ and the thickness after desorption (or the thickness of the sole brush *γ*_brush_): *γ*_ads_ = *γ*_total_ − *γ*_brush_. Values for *γ*_ads_ were found around 75 to 80 Å and are almost twice higher than *γ*_brush_. Moreover, the same results were obtained with P(NIPAM-*co*-MADAP) solutions at various concentrations: 0.05, 0.1, and 1 wt %.

**Figure 7:**
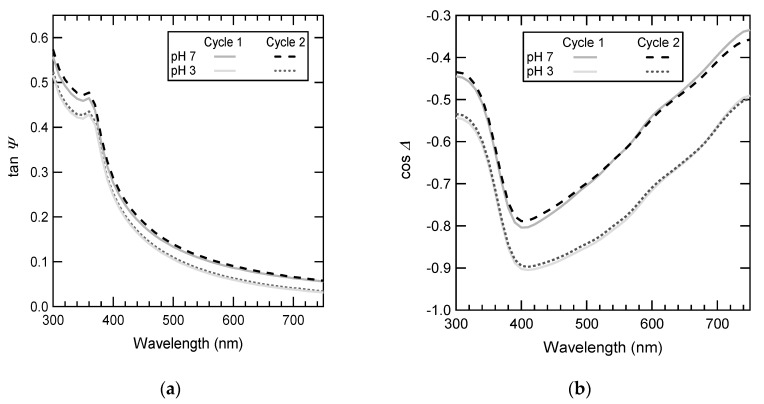
Spectroscopic variation of (**a**) tan Ψ and (**b**) cos Δ. The full lines are from the measurements of the first cycle, and the dotted (pH 3) and dashed (pH 7) lines are from the measurements of the second cycle (and the next ones).

Figure 7 shows the spectroscopic variation of tan Ψ and cos Δ for immersed measurements. The curves measured with the P(NIPAM-*co*-MADAP) solution at pH 7 and in water at pH 3 are clearly different. The thickness *L*_total_ and the refraction index of the polymer layer after adsorption (about 190 Å and 1.40) and desorption (about 95 Å and 1.39) were obtained by fitting the ellipsometry results. From the refraction index, the mean volume fraction of the polymer layer *ϕ*_total_ was estimated: 0.37 after adsorption and 0.32 after desorption. Finally, the dry thickness of the layer *γ*_total_ (and the corresponding amount per unit area) was obtained with the relation: *γ*_total_ = *L*_total_ × *ϕ*_total_. The values of 70 and 30 Å were obtained after adsorption and desorption, respectively.

The values obtained after desorption in the dry state and immersed are comparable even if the first one is slightly higher. This minor discrepancy could be explained by the fact that the PAA brush remains partly hydrated in the dry state since the measurements were not achieved under controlled atmosphere, leading to a slightly overestimated dry thickness.

However, if the values found after desorption are comparable, those obtained after adsorption are significantly different (70 Å in immersion and 120 Å in dry state). An explanation would be an additional coating of copolymer chains on the PAA brush. Among the copolymer chains which were measured in the dry state, one part was strongly adsorbed on the brush while the other part could be removed by the rinsing solution at pH 7. In that case, neutron reflectivity measurements are particularly useful for the comparison. They provide additionally the density profile of the adsorbed chains.

### 3.6. Density Profiles of PAA Brushes with the Adsorbed Copolymers

**Figure 8:**
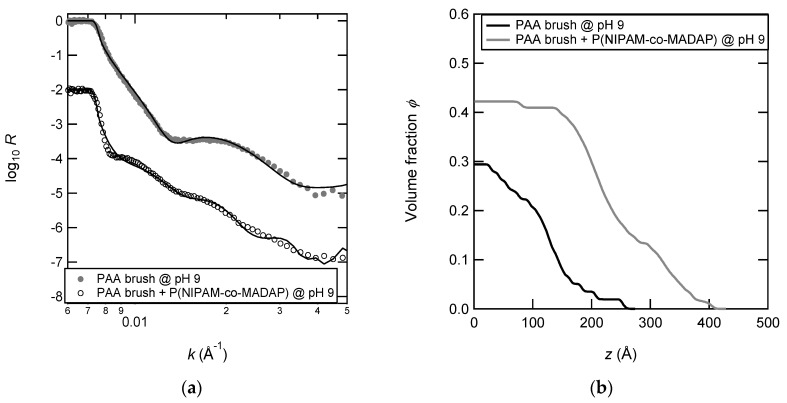
Neutron reflectivity curves (**a**) and polymer density profiles (**b**) corresponding to the best fit of the reflectivity data. The profiles of the poly (acrylic acid) (PAA) brush are shown with (grey line) and without (black line) adsorbed copolymers at pH 9. The PAA brush has these following characteristics: *N* = 328, *γ* = 37 Å, *σ* = 0.125 nm^−2^.

The density profiles of poly (acrylic acid) (PAA) brushes were determined using neutron reflectivity. Figure 8 shows the neutron reflectivity data and the density profiles of the PAA brush at pH 9 in the presence of the solution of P(NIPAM-*co*-MADAP) copolymer. The reflectivity curve of the PAA brush with the adsorbed copolymer displays attenuated Kiessig fringes. A thicker layer and a sharper interface are then expected, which is confirmed by the corresponding profile of the volume fraction of monomers that best fits the reflectivity data. The layer is more stretched with the adsorbed copolymer (*L*_max_ ~ 400 Å) than without it (*L*_max_ ~ 250 Å). The layer with the adsorbed copolymer is also denser near the surface with a volume fraction of 0.42 against 0.30 for the sole PAA brush. This increase of the volume fraction near the surface suggests interpenetration of the P(NIPAM-*co*-MADAP) copolymer inside the PAA brush. It means that the adsorption of the copolymer does not occur only at the border of the brush but there is complexation of the P(NIPAM-*co*-MADAP) chains with the PAA chains inside the brush. Nevertheless, this interpretation is only valid if the density profile of the brush is not modified in the presence of the adsorbed copolymer.

**Figure 9:**
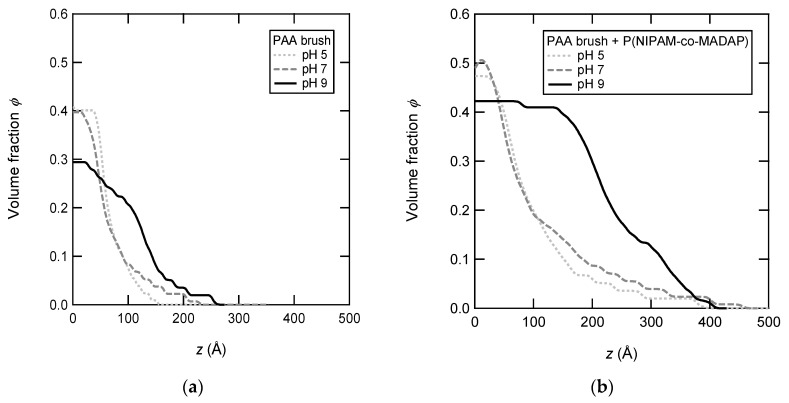
(**a**) Density profiles of a PAA brush (*N* = 328, *γ* = 37 Å, *σ* = 0.125 nm^−2^) without adsorbed copolymers and (**b**) with adsorbed copolymers at various pH: pH 5 (dotted line), 7 (dashed line), and 9 (full line).

The density profiles of the PAA brush in the presence of P(NIPAM-*co*-MADAP) copolymer at various pH are shown in Figure 9. The shape of the density profiles at pH 5 and 7 is similar and is significantly different from the one at pH 9. The profile at pH 9 displays a dense layer broadened on a range of 200 Å. From the density profiles, some quantitative analyses on the adsorption can be carried out. The dry thickness of the adsorbed layer, *γ*_ads_ which is also the amount of adsorbed copolymer per unit area can be deduced: *γ*_ads_ = *γ*_total_ − *γ*. The swollen thickness of the adsorbed layer, *L*_ads_ can also be calculated: *L*_ads_ = *L*_total_ − *L* if the density profile of the brush is supposed to be unmodified in the presence of the adsorbed copolymer. At pH 7, the values of *γ*_ads_ and *L*_ads_ are 30 and 66 Å. They are in good agreement with those measured by ellipsometry in the immersion state. The adsorption of copolymer is twice larger at pH 9 than at 7 (or pH 5), *γ*_ads_ and *L*_ads_ are 68 and 119 Å at pH 9. This result is explained by the high amount of electrostatic charges on the chains: at pH 9, the PAA chains are completely ionized [39], resulting in numerous sites available for adsorption, and the charge neutralization of the PAA chains requires a higher number of counterions—borne by the P(NIPAM-*co*-MADAP) copolymer. Moreover, the ratio of the adsorbed amount which is helpful for quantitative analysis is defined by Equation (5), with *ρ*_copo_ and *ρ*_brush_ the density of P(NIPAM-*co*-MADAP) (*ρ*_copo_ = 1.38 g cm^−3^) and PAA (*ρ*_brush_ = 1.08 g cm^−3^), *M*_copo_ and *M*_brush_ the average molar mass of P(NIPAM-*co*-MADAP) monomers (*M*_copo_ = 119 g mol^−1^) and of PAA monomers (*M*_brush_ = 72 g mol^−1^). The value of *R*_ads_ is equal to 1.43 at pH 9 and 0.7 at pH 7. Actually, it is not surprising that the adsorbed ratio is higher than 1 at pH 9. On the one hand, the opposite charges of the P(NIPAM-*co*-MADAP) copolymer and the PAA brush are not all involved in the complexation, and some counterions are still present in the adsorbed layer. On the other hand, the effective charge ratio of the P(NIPAM-*co*-MADAP) copolymer and the PAA brush is not taken in account since it is not easy to estimate with pH. However, several units of the same copolymer chain are probably involved in the formation of electrostatic complexes with the brush to allow the adsorption. A sketch can be drawn regarding the density profile. At pH 7, the brush is ionized mainly on its top part while the monomer units buried near the substrate surface remain protonated, as shown by Dong et al. [39] In that case, the complexation occurs mostly with the chains units near the solvent, resulting in the adsorption of the copolymer chains in the external part of the brush.

## 4. Conclusions

The swelling-collapse states and the complexation properties of PNIPAM brushes with temperature were investigated using neutron reflectivity. We found that the brush was swollen below the LCST as expected and the swelling ratio was in very good agreement with classic scaling laws predicted by mean-field theories. The swelling-to-collapse ratio was usefully obtained for wide ranges of grafting density and chain length, indicating that the brushes with long chains and intermediate grafting density had the most efficient temperature-responsiveness with the highest ratio. The complexation of PNIPAM brushes with model macromolecules was voluntarily simplified to hydrophobic (LCST-type) interactions. The adsorption of linear chains and comb-like chains (where active side-chains are chemically attached to an acrylamide neutral backbone) containing NIPAM units on PNIPAM brushes was controlled by temperature. The adsorption was reversible: It was found only above the LCST and there was no adsorption below the LCST. The density profiles determined by neutron reflectivity showed that the adsorption of both linear and comb-like macromolecules occurred on collapsed PNIPAM brushes and was preferentially localized on the top of the collapsed brush. The amounts of adsorbed polymers were much higher than the amounts of adsorbed proteins on PNIPAM brushes, suggesting that the complex adsorption of proteins involves many molecular mechanisms additional to hydrophobic coupling such as electrostatic interactions. It could be a great of interest to quantitatively compare specific interactions using model systems. It is on the topic of specific coupling such as electrostatic and hydrophobic coupling involved in the adsorption of model macromolecules on surfaces modified by model polymer brushes. This work will be reported in a forthcoming publication.

## Figures and Tables

**Table 1:** Characteristics of poly (*N*-isopropylacrylamide) (PNIPAM) brushes synthesized by the “grafting onto” method.

*M*_n_ (g mol^−1^)	% Functionalized Polymer	Reaction Time (h)	*γ*^a^ (Å)	*σ*^b^ (nm^−2^)	*D*^c^ (Å)	2*R*_0_^d^ (Å)	*bN*^1/3 e^ (Å)
121,000	100	144	113	0.064	39	184	56
121,000	100	24	95	0.054	43	184	56
121,000	10	24	59	0.034	55	184	56
121,000	10	1	21	0.012	91	184	56
70,000	100	144	104	0.100	32	140	47
70,000	100	72	88	0.085	34	140	47
70,000	100	24	72	0.069	38	140	47
70,000	10	24	50	0.048	46	140	47
70,000	10	1	19	0.018	74	140	47
11,900	100	144	31	0.217	24	58	26

^a^*γ* is the dry thickness of the PNIPAM brush. ^b^ *σ* is the grafting density calculated using the Equation (S2). ^c^ *D* is the average distance between two grafting sites. ^d^ 2*R*_0_ is the diameter of the PNIPAM chain in unperturbed conditions (Θ-conditions). ^e^ *bN*^1/3^ is the size of the PNIPAM chain in the dry state calculated with *b* = (*M*_0_/*ρ*_PNIPAM_ × *N*_A_)^1/3^ = 5.5 Å (with *M*_0_ = 113 g mol^−1^, the molar mass of the NIPAM repeating unit).

**Table 2:** Characteristics of PNIPAM brushes below (20 °C) and above the Lower Critical Solution Temperature LCST (60 °C).

*M*_n_ (g mol^−1^)	*D* (Å)	(*Nb*^3^)^1/3^ (Å)	(*V*_0_)^1/3^ (Å)	(*V*_F_)^1/3^ (Å)	*L*_20_ (Å)	(*L*_20_*D*^2^)^1/3^ (Å)	$\frac{V_{F}}{L_{20}D^{2}}$	*φ*_20_ = *γ*/*L*_20_	*L*_60_ (Å)	(*L*_60_*D*^2^)^1/3^ (Å)	$\frac{L_{20}}{L_{60}}$	*φ*_60_ = *γ*/*L*_60_
121,000	39	56	148	240	806	107	11.3	0.14	185	66	4.4	0.61
121,000	43	56	148	259	860	117	10.8	0.11	240	76	3.6	0.40
121,000	55	56	148	252	697	128	7.6	0.08	152	77	4.6	0.39
70,000	32	47	113	188	611	86	10.4	0.17	222	61	2.8	0.47
11,900	24	26	47	59	107	40	3.2	0.29	108	40	1.0	0.29

*L*_20_ and *L*_60_ are the respective thickness of polymer brushes at 20 and 60 °C; *L*_20_/*L*_60_ is the swollen/collapsed ratio of PNIPAM chains inside the brush; *φ*_T_ and (*L*_T_*D*^2^)^1/3^ are the volume fraction of the PNIPAM brush at the temperature *T* (°C) and the size of an individual chain, respectively.

**Table 3:** Characteristics of the adsorbed layers of poly (*N*-isopropylacrylamide-*co*-acrylic acid) (P(NIPAM-*co*-AA)) linear copolymers on PNIPAM brushes at 60 °C.

*M*_n_ (g mol^−1^)	*γ* (Å)	*σ* (nm^−2^)	*γ*_total_ (Å)	*L*_total_ (Å)	*γ*_ads_ (Å)	*L*_ads_ (Å)	*R*_ads_
121,000	113	7.76 × 10^−2^	157	376	44	298	0.39
121,000	95	6.55 × 10^−2^	132	349	37	263	0.39
121,000	59	4.07 × 10^−2^	158	374	99	322	1.70
70,000	104	12.4 × 10^−2^	154	354	50	257	0.49
11,900	31	21.7 × 10^−2^	137	309	106	273	3.46

*γ*_total_ is the total dry thickness of the brush with the adsorbed copolymer. *L*_total_ is the total swollen thickness of the brush with the adsorbed copolymer. *γ*_ads_ is the dry thickness of the adsorbed layer (without the brush). *L*_ads_ is the swollen thickness of the adsorbed layer (without the brush). *R*_ads_ is the ratio of amount of adsorbed polymer.

## Supplementary Materials

The following are available online at https://www.mdpi.com/2073-4360/12/1/153/s1. Discussion on the regimes of the PNIPAM-grafted surfaces; determination method of the volume fraction of monomers by neutron reflectivity; swelling ratios of PNIPAM brushes.
