# Supplementary material for: Responsive Adsorption of N-Isopropylacrylamide Based Copolymers on Polymer Brushes
Source: Polymers (Basel). 2020 Jan 7;12(1):153. doi: 10.3390/polym12010153 (PMC7022643; doi:10.3390/polym12010153)
Supplement: Supplementary file 1 [file polymers-12-00153-s001.pdf]

Supporting information

Responsive Adsorption of *N*-isopropylacrylamide Based Copolymers on Polymer Brushes

Guillaume Sudre, Elodie Siband, Bruno Gallas, Fabrice Cousin, Dominique Hourdet, Yvette Tran

Regimes of the PNIPAM-grafted surfaces: brushes or mushrooms?

If the dry layer is considered to contain only polymer chains, the volume occupied by a single chain (Nb^3) can be calculated from the following relation:

$$Nb^3 = \gamma \times D^2 = \frac{\gamma}{\sigma} = \frac{M_n}{\rho_{PNIPAM} \times N_A} \qquad [S1]$$

where D is the average distance between two anchoring sites, σ is the grafting density or the number of chains per unit area, ρ_{PNIPAM} = 1.10 g cm^{-3} is the density of PNIPAM, M_n the number-average molar mass of the polymer, N corresponds to the number of monomers per chain or the degree of polymerization and b^3 is the size of a monomer.

The grafting density can be calculated from the dry thickness measured by ellipsometry and the molar mass M_n determined by SEC:

$$\sigma = \frac{\gamma \times \rho_{PNIPAM} \times N_A}{M_n} \qquad [S2]$$

The distance between two anchoring chains is an important parameter that defines the conformation regime: the "mushroom regime", where D is higher than twice the radius of gyration of a free polymer chain (R_0), and the "brush regime" for $D < 2R_0$. In the brush regime, polymer chains are densely packed so that they have to stretch in the direction

normal to the substrate and cannot take an isotropic conformation as for free chains. Consequently, the scaling laws representing the swelling or collapse of polymer brushes in good or bad solvent conditions are different from those of free polymer chains. In order to calculate the size of a free chain in well-defined solvent conditions we have used the quantitative definitions given for non-solvent (dry state) $bN^{1/3} = \left(M_n / \rho N_A\right)^{1/3}$ and Θ-solvent:

$$R_0 = \left(C_\infty N l^2 / 3\right)^{1/2} \qquad \text{[S3]}$$

where C_∞ = 10 is the characteristic ratio of PNIPAM [1] and l = 1.54 Å, the carbon-carbon bond length.

We have to consider that at 20 °C the PNIPAM chain is in rather good solvent conditions, i.e. well above the Θ-conditions, and the real size of the chain should be higher that its unperturbed dimension. Similarly, in the collapsed state, the PNIPAM chain could keep water molecules in its pervaded volume and its real size should be larger than in the dry state. Nevertheless, even if we consider that the globular chain contains about 50 wt% of water, as we will see later, its size will be only 25% higher compared to the dry state.

Determination method of the volume fraction of monomers by neutron reflectivity

Neutron reflectivity was used to study PNIPAM brushes in water. This technique is sensitive to the scattering length density profile normal to the surface $Nb(z)$ and thus to the profile of the volume fraction of monomers $\phi(z)$. The specular reflection does not allow the distinction between the roughness and the inter-diffusion of a layer if the size of heterogeneities is smaller than the coherent length of neutrons (which is about a few microns). Lateral morphologies of PNIPAM brushes determined by AFM shows that the size of the aggregates is lower than 50 nm in the plane and the layer roughness (precisely the root-mean-square deviation of the surface) is a few nanometers, as also observed by Bittrich et al. [2] It means that the average thickness and the volume fraction profile of PNIPAM brushes can be determined unambiguously in spite of the in-plane irregularities.

Figure 1 shows an example of neutron reflectivity data and the profile of the volume fraction of monomers corresponding to the best fit of the experimental results. For this sample of PNIPAM brush (M_n = 121 kg.mol^{-1} and σ = 0.078 nm^{-2}) measured at 20 °C, the reflectivity curve does not display obvious Kiessig fringes and the density profile is consistently smooth or gradual. The smoothly decaying profile is in agreement with mean-field calculations which predict analytical (parabolic or tanh-derived) functions for the volume fraction profiles [3-6]. According to the step profile used to fit the experimental data, the conformation of chains is unfortunately limited as the chain ends should be at the same distance from the surface. A soft profile provides more flexibility since all the chains, even attached, have not necessary the same stretching and the extremities of chains can be at any distance from the surface. Moreover, the polydispersity of chains can also be taken into account using an additional component (exponential queue for example) to the main parabolic profile. The analytical

forms of the density profile of polymer brushes were investigated in details in previous publications and in particular for poly(acrylic acid) brushes [7]. In the present work, where we aim at comparing the density profiles of polymer brushes at different temperatures, with or without adsorbed polymers, a simple step model is well adapted.

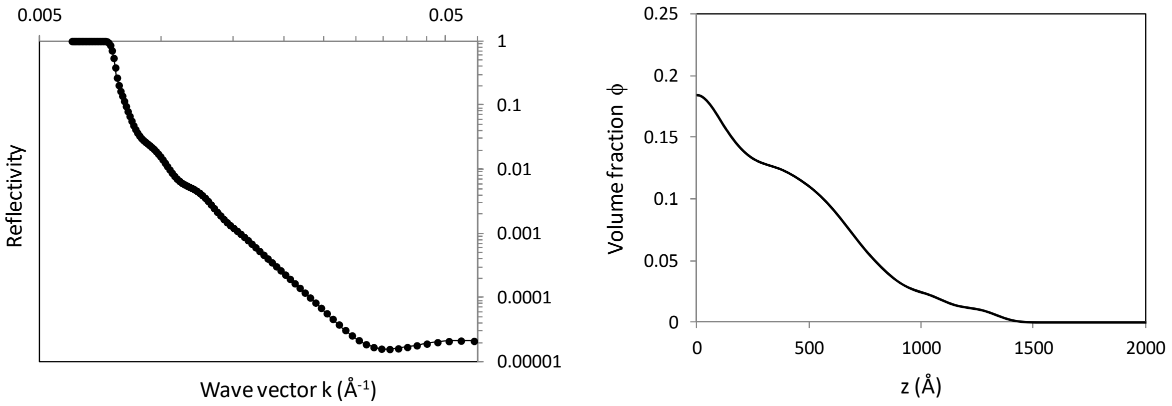

Figure S1. Neutron reflectivity curve (left) and polymer density profile (right) corresponding to the best fit of the reflectivity data. The neutron reflectivity experiment was performed in D_2O at 20 °C. The PNIPAM brush has the following characteristics: M_n = 121 kg mol^{-1}, γ = 113 Å and σ = 0.078 nm^{-2}.

Swelling ratios of PNIPAM brushes

Note that the inverse of the swelling ratio, γ/L, corresponds to the average volume fraction of polymer in the swollen layer. From a theoretical point of view, the swelling ratio of polymer brushes can be estimated on the basis of scaling relations. Starting from equation [S1], a first relation between the dry thickness γ and the grafting density σ can be extracted:

$$\gamma = Nb^3\sigma \qquad [S4]$$

The swollen thickness L of neutral polymer brushes in good solvent is given by Alexander-de Gennes scaling law [8-10] :

$$L \propto Nb^{5/3}\sigma^{1/3} \qquad [S5]$$

The same argument will give $L \propto Nb^2\sigma^{1/2}$ in Θ-conditions and $L \propto Nb^3\sigma$ in bad solvent conditions. Finally, the swelling ratio can be obtained by coupling equations [S4] and [S5]:

$$\frac{L}{\gamma} \propto N^0\sigma^{\beta} \qquad [S6]$$

with β = -2/3, -1/2 and 0 for good, Θ- and bad solvents, respectively.

Note that equation [S6] is strictly valid if the ratio of the monomer size in the swollen brush to that in the dry brush is independent of the grafting density. This condition was met for all the brushes in the range of grafting density investigated. As proof, the distance between two attached chains (D = 17 Å for the lowest) is in all cases much higher than the monomer size (b = 5 Å).

Accordingly, experimental swelling ratios obtained with polymer brushes of different molar masses have been plotted in **Figure S2** *versus* their grafting density.

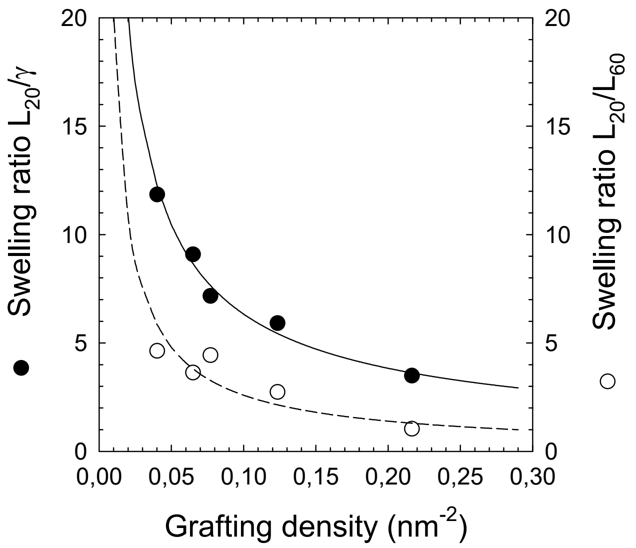

Figure S2. Swelling ratio of PNIPAM brushes at 20 °C (L_{20}/γ; ●) and swollen/collapsed ratio (L_{20}/L_{60}; ○) as a function of the grafting density. The data are best fitted with a power law with an exponent of -0.69 for L_{20}/γ and -0.89 for L_{20}/L_{60}.

Contrary to the swelling ratio (L_{20}/γ), no physical meaning is expected for the scaling exponent of L_{20}/L_{60} versus the grafting density as this variation takes into account the fraction of water in the brush which varies with the molar mass of PNIPAM chains. Indeed, the average volume fraction of water in the collapsed brushes ($1-\phi_{60}$; see **Table 2**) varies from 40 to 50 % for brushes prepared with M_n = 70–121 kg mol^{-1} and up to 70 % for the brush grafted with M_n = 11.9 kg mol^{-1}. It is also worth mentioning that in this last case, the thickness and then the swelling of the polymer brush remains almost unchanged between 20 and 60 °C (no deswelling).

Retrospectively, it is now possible to calculate the size of free PNIPAM chains at 20 °C as we know the thickness of the swollen layer and we have shown that water is a good solvent at this temperature. For that purpose we will consider that each polymer chain inside the brush self-organize into an array of correlation blobs perpendicular to the surface. If g is the number of monomers per correlation blob and N the total number of monomer per chain, we have in good solvent conditions:

$$D^3 = A \times g^{9/5} \text{ [10]} \quad \text{and} \quad V_F = A \times N^{9/5} \qquad \text{[S7]}$$

where A is a common prefactor and $V_F^{1/3}$ is the Flory radius, i.e. V_F is the pervaded volume of an isolated polymer chain in good solvent conditions. As the number of correlation blobs (N/g) can be calculated from the swollen thickness of the polymer brush ($N/g=L/D$), then it comes:

$$V_F = L^{9/5} D^{6/5} \qquad \text{[S8]}$$

As expected from theoretical predictions (equations [S3] and [S8]), we can see in **Table S1** that free PNIPAM chains swell in good solvent ($V_F^{1/3}$) compared to Θ-conditions ($V_0^{1/3}$) and that the level of swelling $(V_F/V_0)^{1/3}$ increases with the molar mass. We can also notice that the Flory volume calculated from the three polymer layers prepared with the PNIPAM of higher molar mass are almost the same which confirms the reliability of the experiments and the overall data treatment. From the Flory volume, which gives information about the size of the chain in good solvent and unconfined conditions, the comparison with the volume occupied by the same chain in the polymer brush tell us about the level of overlapping of confinement inside the swollen layer. We can see in **Table S1** that at 20 °C the level of chain overlapping (V_F/D^2L) is always higher than 1 and that PNIPAM chains are in the brush regime for all the samples as previously discussed. Contrary to the polymer volume fraction ($\varphi = \gamma/L \propto N^0\sigma^{2/3} \propto N^0D^{-4/3}$) that only depends on the grafting density, the level of chain overlapping clearly emphasizes the impact of the molar mass and the parallel that can be drawn between the brush regime and semi-dilute polymer solutions.

M_n (g mol^{-1})	D (Å)	L_{20} (Å)	$(Nb^3)^{1/3}$ (Å)	$(L_{20}D^2)^{1/3}$ (Å)	$(V_F)^{1/3}$ (Å)	$(V_0)^{1/3}$ (Å)	$V_F/L_{20}D^2$	$\phi_{20} = \gamma/L$
121 000	39	806	56	107	240	148	11.3	0.14
121 000	43	860	56	117	259	148	10.8	0.11
121 000	55	697	56	128	252	148	7.6	0.08
70 000	32	611	47	86	188	113	10.4	0.17
11 900	24	107	26	40	59	47	3.2	0.29

Table S1. Characteristic sizes of PNIPAM chains at 20 °C inside the brush $(L_{20}D^2)^{1/3}$ and calculated values for the same free PNIPAM chains in the dry state ($N^{1/3}b$), in Θ-conditions ($V_0^{1/3}$) and in good solvent $V_F^{1/3}$. $V_F/L_{20}D^2$ is the overlapping ratio of PNIPAM chains inside the brush and φ_{20} their volume fraction.

1. Kubota, K.; Hamano, K.; Kuwahara, N.; Fujishige, S.; Ando, I. Characterization of Poly(N-isopropylmethacrylamide) in Water. *Polymer Journal* **1990**, *22*, 1051-1057, doi:10.1295/polymj.22.1051.
2. Bittrich, E.; Burkert, S.; Muller, M.; Eichhorn, K.J.; Stamm, M.; Uhlmann, P. Temperature-Sensitive Swelling of Poly(N-isopropylacrylamide) Brushes with Low Molecular Weight and Grafting Density. *Langmuir* **2012**, *28*, 3439-3448.
3. Milner, S.T.; Witten, T.A.; Cates, M.E. A Parabolic Density Profile for Grafted Polymers. *Europhys Lett* **1988**, *5*, 413-418.
4. Milner, S.T.; Witten, T.A.; Cates, M.E. Effects of Polydispersity in the End-Grafted Polymer Brush. *Macromolecules* **1989**, *22*, 853-861.
5. Skvortsov, A.M.; Pavlushkov, I.V.; Gorbunov, A.A. On the Structure of a Monolayer of Grafted Polymer-Chains. *Vysokomol Soedin a+* **1988**, *30*, 503-508.
6. Skvortsov, A.M.; Pavlushkov, I.V.; Gorbunov, A.A.; Zhulina, Y.B.; Borisov, O.V.; Pryamitsyn, V.A. Structure of Dense-Grafted Polymer Monolayers. *Vysokomol Soedin a+* **1988**, *30*, 1615-1622.
7. Sudre, G.; Hourdet, D.; Creton, C.; Cousin, F.; Tran, Y. pH-Responsive Swelling of Poly(acrylic acid) Brushes Synthesized by the Grafting Onto Route. *Macromol. Chem. Phys.* **2013**, *214*, 2882-2890, doi:10.1002/macp.201300477.
8. Alexander, S. Adsorption of Chain Molecules with a Polar Head a-Scaling Description. *J Phys-Paris* **1977**, *38*, 983-987.
9. De Gennes, P.G. Scaling Theory of Polymer Adsorption. *J Phys-Paris* **1976**, *37*, 1445-1452.
10. De Gennes, P.G. Conformations of Polymers Attached to an Interface. *Macromolecules* **1980**, *13*, 1069-1075.
